# Rituximab in myositis: where are we now? A survey of current usage

**DOI:** 10.1093/rheumatology/kead010

**Published:** 2023-01-09

**Authors:** Tatiana Oliveira, Reşit Yıldırım, Claire Deakin, David Isenberg

**Affiliations:** Internal Medicine Unit, Department of Medicine, Hospital de Cascais, Cascais, Portugal; Division of Rheumatology, Department of Internal Medicine, Eskişehir Osmangazi University, Turkey; Centre for Rheumatology, Department of Medicine, University College London, London, UK; Centre for Rheumatology, Department of Medicine, University College London, London, UK

Rheumatology key message

- Rituximab appears to be an effective treatment option in myositis patients.

Dear Editor, In a retrospective analysis 7 years ago, it was estimated that ∼78% of patients with inflammatory myositis were considered to have improved with rituximab (RTX) [1]. The International Myositis Assessment & Clinical Studies (IMACS) group felt it was timely to undertake a prospective study of the use of RTX in myositis. Members of the group were invited to send information about RTX-treated patients from July 2021 during the following year. After lengthy discussions, a simple proforma was produced and sent to the IMACS members on four occasions during the year. The key aims of the study were to: assess the current use of B cell depletion in patients with myositis; obtain background information about which particular patients are being treated in this way; and ascertain if B cell depletion continues to be effective in myositis.

Fifty myositis patients from nine referring centres were distinguished in two groups based on the time of first RTX infusion (29 had been prescribed it before July 2021, designated previous users, and 21 were new starters). The patients’ demographic features, characteristics of myositis and treatment response are summarized in Table 1. The median time from the diagnosis until the first RTX infusion was 21.5 months (interquartile range = 65). Most were females (74%), of Caucasian origin (56%) and had a diagnosis of anti-synthetase syndrome (56%). Three out of 50 patients were myositis-specific antibody (MSA) negative. The most common immunosuppressive agent used with RTX was CS (84%), followed by MMF (36%), MTX (10%), AZA (8%), CSA (8%) and CYC (2%). RTX was preferred as a first line drug with steroids in five patients (two were reported to have responded well). Effectiveness was assessed by the treating physician on the basis of clinical observation, reduction in the creatine kinase and capacity to reduce steroids. Based on these components, RTX was reported to be effective in 43 patients (86%), and no benefit was seen in 7 patients (14%). Treatment response was found to be similar in both groups (89.7% *vs* 81%, *P* = 0.434). Of note, no statistically significant difference was seen in RTX effectiveness among types of myositis (anti-synthetase syndrome *vs* others), MSAs and genders. Steroid taper was successfully achieved in 78.7% of patients. Adverse events were noted in seven patients; all were infectious complications with requirement for hospitalization in three cases. Three minor side effects including two infusion reactions and one nausea were reported during the entire follow-up.

**Table 1.** Patients’ demographics data, myositis features and rituximab response assessment

	Frequency (%)
Gender	
Female	37 (74)
Male	13 (26)
Ethnicity origin	
Caucasian	28 (56)
East-Asian	16 (32)
South-Asian	5 (10)
Afro-Caribbean	1 (2)
Organ involvement	
Muscle	46 (92)
Lungs	35 (70)
Skin	31 (62)
Skeletal	22 (44)
Heart	6 (12)
Gastrointestinal	3 (6)
Type of myositis	
ASS	28 (56)
DM	12 (24)
IMNM	5 (10)
PM	4 (8)
OM	1 (2)
Rituximab response assessment	
Muscle symptoms improvement (*n* = 37)	34 (92)
Respiratory symptoms improvement (*n* = 34)	25 (74)
Skin symptoms improvement (*n* = 21)	20 (95)
Skeletal symptoms improvement (*n* = 17)	14 (82)
CK decreasing (*n* = 28^a^)	26 (93)
Tapering steroids (*n* = 47^b^)	37 (79)

a Eighteen patients had normal CK at the time of first RTX use, CK was not measured in two and not available in two.

b Two patients were not on steroids at the time of first RTX use, data were not available in one. ASS: anti-synthetase syndrome; CK: creatine kinase; IMNM: immune-mediated necrotizing myopathy; OM: overlap myositis.

Despite the paucity of well-designed randomized controlled trials, the evidence of prominent B lymphocyte infiltration in the muscles of patients and circulating autoantibodies in the majority of idiopathic inflammatory myopathies (IIM) has suggested that B cell depleting therapy might be efficacious in IIM [2]. In the RIM (Rituximab in Myositis) trial [3], 83% of refractory IIM patients were found to improve with RTX treatment, although no difference in efficacy between early *vs* late (8 weeks later) treatment groups was seen in primary and secondary endpoints. However, the trial design was later criticized as the 8-week interval of the placebo phase, based on ethical considerations, may have been too short to detect a significant difference [4]. Our observational data suggests that there is no change in RTX effectiveness whether it was given for the first time or as a repeat set of infusions and the overall response rate in our study was similar to previous reports.

Recent advances in the identification of novel autoantibodies in myositis have led to a better understanding regarding the disease's nature, response to treatment, and prognosis [5]. Subgroup analysis according to MSA status in RIM trial revealed that those with a positive MSA (especially anti-Jo1 or anti-Mi-2 positive subsets) had a better clinical improvement following B cell depletion therapy compared with the negative autoantibody group, conferring MSA status as a major predictive factor for clinical improvement [6]. Given the limited number of patients who were mostly MSA positive in our study group, we compared anti-synthetase-positive and -negative groups in terms of RTX efficacy, but found no difference. Furthermore, the treatment response was similar between genders.

While steroids are invariably used in IIM and are effective in the majority of patients [7], sustained response cannot always be achieved due to drug-related complications and relapses following taper. RTX has been considered to be a promising drug having the potential to reduce the steroid dose over the long term, albeit disappointing results were reported in a meta-analysis of lupus trials [8]. In contrast, retrospective studies in refractory myositis have shown that RTX may be an effective option as a steroid-sparing agent [3, 9]. Our results support this observation. Heterogeneous distribution, difference in response assessment among referring centres and loss of some data from the retrospective part (especially steroid dose) are limitations of our study.

In conclusion, although supportive clinical trials are lacking, the data we now present, together with an earlier report, does encourage the belief that B cell depletion is a viable treatment option when managing myositis.

## Data Availability

The authors would be happy to respond to any reasonable request for the data from which this letter was made possible.
